# Lymphoma encasing the heart: large mediastinal mass with myocardial infiltration and coronary artery involvement

**DOI:** 10.1007/s10554-025-03496-6

**Published:** 2025-08-30

**Authors:** Taraneh Aziz-Safaie, Alexander M. C. Böhner, Julian A. Luetkens, Alexander Isaak

**Affiliations:** 1 Department of Diagnostic and Interventional Radiology, University Hospital Bonn, Venusberg-Campus 1, 53127 Bonn, Germany; 2 Quantitative Imaging Laboratory Bonn, Bonn, Germany

**Keywords:** Mediastinal neoplasms, Lymphoma, B-cell, Coronary vessels, Magnetic resonance imaging, Computed tomography angiography

## Abstract

Primary mediastinal B-cell lymphoma with myocardial infiltration and coronary encasement is a rare cause of chest pain and dyspnea in young patients. Cardiac magnetic resonance imaging and photon-counting coronary CT play a pivotal role in detecting myocardial and coronary artery involvement. Early recognition is crucial as these findings directly affect treatment and prognosis.

## Case information

A 29-year-old male was referred to cardiology with recurrent pulmonary infections over four months. He reported thoracic pressure and dyspnea. Transthoracic echocardiography showed pericardial effusion without right heart strain and normal left ventricular function. ECG was unremarkable. Inflammatory markers, Troponin T, Pro-BNP, and LDH were elevated. Given the patient’s age, clinical presentation, pericardial effusion, and inflammatory markers, perimyocarditis was suspected.

**Fig. 1:**
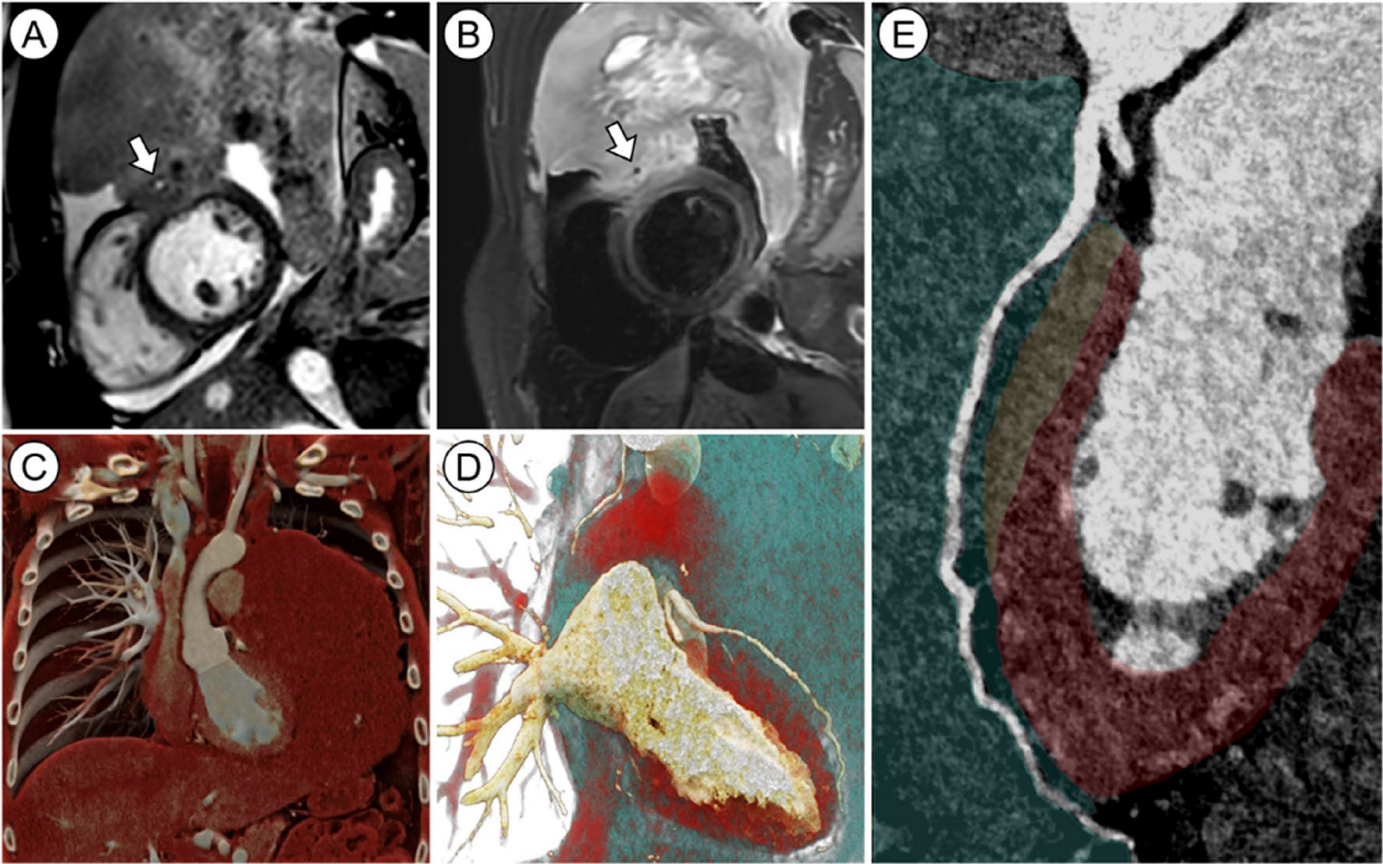
.

Cardiac magnetic resonance imaging (CMR) on the day of admission revealed a large, centrally necrotic mediastinal mass extending from the upper mediastinum to the diaphragm (Fig. 1A–C), with anterior myocardial infiltration (Fig. 1A, B), pericardial effusion (Figure A), and encasement of the left anterior descending coronary artery (Fig. 1A, B, arrows). No postischemic scar was detected. Staging CT including ECG-gated coronary CT (Fig. 1D, E) ruled out high-grade coronary stenosis and showed no other lesions or lymphadenopathy. Differential diagnoses included mediastinal lymphoma or, less likely, a germ cell tumor. Ultrasound-guided biopsy confirmed primary mediastinal B-cell lymphoma. Prednisolone pre-phase therapy was initiated.

This case highlights the importance of CMR as a gatekeeper for rare causes of atypical chest pain. Myocardial infiltration in mediastinal lymphoma is rare and coronary encasement is even rarer. These findings, however directly impact treatment and prognosis.

## Data Availability

No datasets were generated or analysed during the current study.

